# Efficacy of empagliflozin in patients with metabolic dysfunction-associated steatotic liver disease with or without diabetes: a systematic review and meta-analysis of randomized controlled trials

**DOI:** 10.3389/fmed.2025.1712586

**Published:** 2026-02-04

**Authors:** Khalid I. AlHussaini

**Affiliations:** Department of Internal Medicine, College of Medicine, Imam Mohammad Ibn Saud Islamic University (IMSIU), Riyadh, Saudi Arabia

**Keywords:** empagliflozin, metabolically-dysfunction-associated steatotic liver disease, liver enzymes, fibrosis, SGLT2 inhibitors, meta-analysis

## Abstract

**Background:**

Empagliflozin, a sodium-glucose cotransporter-2 (SGLT2) inhibitor, has demonstrated potential hepatic benefits in patients with metabolic dysfunction-associated steatotic liver disease (MASLD), particularly among those with type 2 diabetes mellitus (T2DM). This meta-analysis aimed to evaluate the efficacy of empagliflozin on hepatic and metabolic outcomes in patients with MASLD.

**Methods:**

A systematic literature search of PubMed, Scopus, and Web of Science was conducted up to September 2025 to identify randomized controlled trials (RCTs) evaluating empagliflozin in MASLD patients with or without T2DM. Primary outcomes included changes in liver enzymes including alanine aminotransferase (ALT) and aspartate aminotransferase (AST), hepatic steatosis and fibrosis indices including controlled attenuation parameter (CAP), liver stiffness measurement (LSM), aspartate aminotransferase to platelet ratio index (APRI), fibrosis-4 index (FIB-4), and the MASLD fibrosis score (NFS), and secondary outcomes included lipid parameters, glycemic control, and anthropometric measures.

**Results:**

Eight RCTs involving 672 participants (353 empagliflozin and 319 placebo) were included. Empagliflozin significantly reduced ALT (mean difference [MD] = −9.36, 95% CI: −16.07 to −2.66, *p* = 0.006) and AST (MD = −9.09, 95% CI: −15.41 to −2.78, *p* = 0.005) compared to placebo. A significant reduction was also observed in triglyceride levels (MD = −29.29, 95% CI: −53.14 to −5.45, *p* = 0.02). No significant differences were found for CAP (MD = −5.72, *p* = 0.29), LSM (MD = −0.49, *p* = 0.38), APRI (MD = −0.02, *p* = 0.36), FIB-4 (MD = −0.06, *p* = 0.34), or NFS (MD = −0.04, *p* = 0.83). Similarly, no significant effects were observed for body weight, body mass index (BMI), fasting blood sugar, or glycated hemoglobin (HbA1c).

**Conclusion:**

Empagliflozin is associated with significant improvements in liver enzyme levels and a reduction in triglyceride levels in patients with MASLD. However, no clear benefit was observed in non-invasive markers of hepatic steatosis or fibrosis. Further large-scale, long-duration RCTs with histological endpoints are needed to confirm these findings and establish empagliflozin’s role in MASLD management.

## Introduction

Metabolic-dysfunction-associated steatotic liver disease (MASLD) is a common comorbidity in individuals with type 2 diabetes mellitus (T2DM), affecting about 38% globally (1–4). Both conditions are closely linked through a shared pathophysiological mechanism—insulin resistance—which not only underpins MASLD but also contributes to the increased likelihood of developing T2DM (5, 6). The advanced stage of MASLD, referred to as metabolic dysfunction-associated steatohepatitis (MASH), is characterized by hepatic fat accumulation exceeding 5% of liver volume along with elevated liver enzymes such as alanine aminotransferase (ALT) and aspartate aminotransferase (AST) (7). However, in individuals with T2DM, ALT levels alone may not accurately reflect the severity of hepatic involvement (8). As MASH advances, it can lead to significant liver complications, including fibrosis, cirrhosis, and hepatocellular carcinoma (HCC), thereby increasing the risk of liver-related mortality (9, 10) as well as cardiovascular morbidity (11).

Empagliflozin, a member of the sodium-glucose cotransporter-2 (SGLT2) inhibitor class, is an oral antidiabetic agent that has become a key component in the management of T2DM. It acts by blocking SGLT2 receptors in the renal proximal tubules, which decreases glucose reabsorption and enhances urinary glucose excretion, thereby lowering blood glucose concentrations (12). Beyond its primary glycemic effects, empagliflozin has demonstrated additional metabolic benefits, including body weight reduction, decreased blood pressure, and favorable cardiovascular outcomes, as shown in major trials such as EMPA-REG OUTCOME (13). Emerging evidence also suggests potential hepatic benefits of empagliflozin in patients with MASLD and MASH, although the exact mechanisms behind these effects remain to be fully elucidated (14, 15).

Despite encouraging preliminary findings, the role of empagliflozin in the treatment of MASLD is still under investigation. While some clinical trials report improvements in liver biochemistry and other hepatic markers with SGLT2 inhibitor therapy, others showed no or inadequate efficacy (5). These disparities may stem from differences in patient selection, study methodologies, or the varying stages of liver disease examined. Consequently, a systematic evaluation of the current literature is essential to determine the efficacy of empagliflozin on hepatic outcomes, particularly its effects on liver enzyme levels, fat accumulation, fibrosis, and hepatic inflammation in patients with MASLD (16).

Therefore, this study aims to systematically evaluate the available evidence on the therapeutic effects of empagliflozin in patients with MASLD, with or without T2DM. Specifically, this meta-analysis seeks to assess the impact of empagliflozin on liver enzyme levels, particularly ALT and AST; to examine its effects on non-invasive markers of hepatic steatosis and fibrosis such as controlled attenuation parameter (CAP), liver stiffness measurement (LSM), aspartate aminotransferase to platelet ratio index (APRI), fibrosis-4 index (FIB-4), and the MASLD fibrosis score (NFS); and to investigate changes in metabolic parameters including lipid profile (triglycerides, HDL, and LDL), body weight, body mass index (BMI), fasting blood sugar (FBS), and hemoglobin A1c (HbA1c).

## Methods

### Search strategy

A systematic literature search was conducted in accordance with PRISMA guidelines (17) to identify randomized controlled trials (RCTs) evaluating the effects of empagliflozin in patients with MASLD, with or without T2DM. Electronic databases including PubMed, Scopus, and Web of Science that were searched from inception to September 2025, using a combination of keywords and MeSH terms: (“empagliflozin”) AND (“non-alcoholic fatty liver disease” OR “MASLD” OR “non-alcoholic steatohepatitis” OR “NASH” OR “Metabolic dysfunction-associated steatotic liver disease” OR “Metabolic dysfunction-associated steatohepatitis” OR “MASH” OR “NAFLD”). No language or date restrictions were applied. Reference lists of retrieved articles were manually screened to identify additional relevant studies.

### Eligibility criteria and screening

Studies were eligible for inclusion if they met the following criteria:

- Population: Adults diagnosed with MASLD, with or without T2DM.
- Intervention: Empagliflozin.
- Comparator: Placebo or standard care.
- Outcomes: At least one of the following: liver enzyme levels (ALT and AST), hepatic steatosis or fibrosis indices (CAP, LSM, APRI, NFS, and FIB-4), lipid parameters (triglycerides, HDL, and LDL), anthropometric measures (body weight and BMI), or glycemic control markers (FBS and HbA1c).
- Design: RCTs.

Exclusion criteria included studies investigating other drugs or diseases, different outcomes, reviews, case reports, editorials, abstracts and comments. The screening of titles and abstracts was conducted first. Full-text articles were retrieved for studies that met the inclusion criteria or for which eligibility could not be determined from the abstract alone.

### Data extraction

Data were extracted using a standardized form. Extracted information included study ID, design, country, population characteristics, sample size, mean age and standard deviation, gender distribution, and baseline features for both intervention and control groups. The following outcome measures were extracted post-intervention for meta-analysis: liver enzymes (ALT and AST), liver stiffness and fat indices (CAP, LSM, APRI, NFS, and FIB-4), lipid parameters (triglycerides, HDL, and LDL), body weight, BMI, and glycemic markers (FBS and HbA1c). Where data were not directly reported, corresponding authors were contacted, or values were estimated using appropriate methods (e.g., from graphs or medians where applicable). Corresponding authors were also contacted for missing data; otherwise, inputation methods were applied appropriately.

### Risk of bias assessment

The risk of bias in the included studies was evaluated using the Cochrane Risk of Bias 2.0 (RoB 2) tool (18), which assesses five key domains: the randomization process, deviations from intended interventions, missing outcome data, outcome measurement, and selection of reported results. Each domain was categorized as presenting a “low risk,” “some concerns,” or a “high risk” of bias.

### Statistical analysis

Statistical analysis was performed using RevMan software. For continuous variables, mean differences (MDs) along with 95% confidence intervals (CIs) were calculated to estimate pooled effects. A random-effects model was employed to account for variations in study design and population characteristics. Between-study heterogeneity was assessed using the I^2^ statistic, with thresholds of 25, 50, and 75% representing low, moderate, and high heterogeneity, respectively. A *p* ≤ 0.05 was considered indicative of statistical significance. A qualitative synthesis was conducted for three studies that did not include eligible data for the analysis.

## Results

### Searching and screening

**Figure 1:**
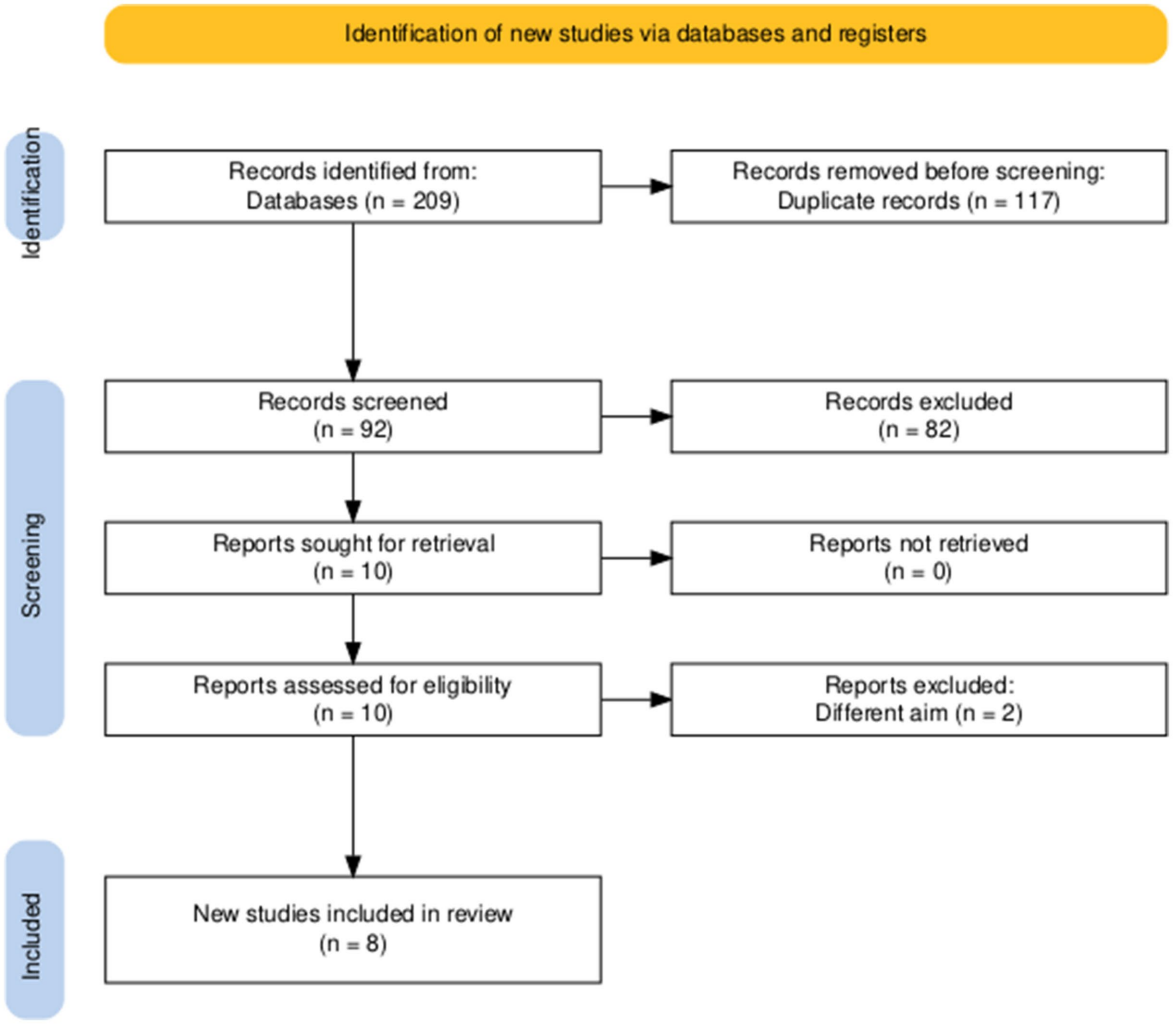
PRISMA flow diagram of searching and screening processes.

The search process initially identified 209 records, of which 117 were removed as duplicates. The remaining 92 articles underwent title and abstract screening, resulting in the selection of 10 studies for full-text assessment. Following a thorough eligibility evaluation, eight studies met the inclusion criteria and were incorporated into the final systematic review and meta-analysis (5, 16, 19–24) (Figure 1).

### Baseline characteristics

The meta-analysis included eight RCTs, assessing the effects of empagliflozin in patients with MASLD, including those with and without T2DM. The studies collectively enrolled a total of 672 participants: 353 in the empagliflozin (intervention) groups and 319 in the placebo (control) groups. The sample sizes of individual studies ranged from 42 to 119 participants. Four of the eight studies were conducted in Iran, while one study was from India, China, the USA, and Germany. The study populations varied slightly in disease profile; two studies included patients with MASLD without diabetes, one study included patients with and without diabetes, while five studies focused on patients with both MASLD and diabetes or MASH with diabetes. The mean age across studies ranged from 43.8 to 61.5 years. Gender distribution varied among studies. The proportion of male participants in the intervention arms ranged from 42.9 to 69%, while in the control groups it ranged from 37.8 to 69% (Table 1).

**Table 1:** Baseline characteristics of the included studies.

Study ID	Design	Country	Population	Sample size		Age, mean (SD)		Male, *n* (%)	
				Intervention	Control	Intervention	Control	Intervention	Control
Taheri 2020 (21)	RCT	Iran	MASLD and no diabetes	43	47	43.8 (9.7)	44.1 (9.3)	28 (65.1)	22 (46.8)
Chehrehgosha 2021 (5)	RCT	Iran	MASLD and diabetes	35	37	50.5 (8.4)	51.8 (7.8)	15 (42.9)	14 (37.8)
Kuchay 2018 (16)	RCT	India	MASLD and diabetes	22	20	NR	NR	NR	NR
Mehr 2025 (19)	RCT	Iran	MASH and diabetes	55	55	50 (14)		56 (49.1)	
Shojaei 2025 (20)	RCT	Iran	MASLD and diabetes	69	50	46.32 (8.11)	52.56 (10.26)	38 (54.29)	32 (45.71)
Cheung 2024 (23)	RCT	China	MASLD and no diabetes	49	49	56.3 (10.2)	55.8 (10.3)	27 (55.1)	27 (55.1)
Abdelgani 2024 (22)	RCT	USA	MASLD with and without diabetes	38	19	49 (2.5)	52 (2.5)	20 (52.6)	10 (52.6)
Kahl 2020 (24)	RCT	Germany	MASLD and diabetes	42	42	62.7 (7)	61.5 (10)	29 (69)	29 (69)

MASLD, metabolic dysfunction-associated steatotic liver disease; MASH, metabolic dysfunction-associated steatohepatitis; SD, standard deviation; NR, not reported; RCT, randomized controlled trial.

### Risk of bias assessment

**Figure 2:**
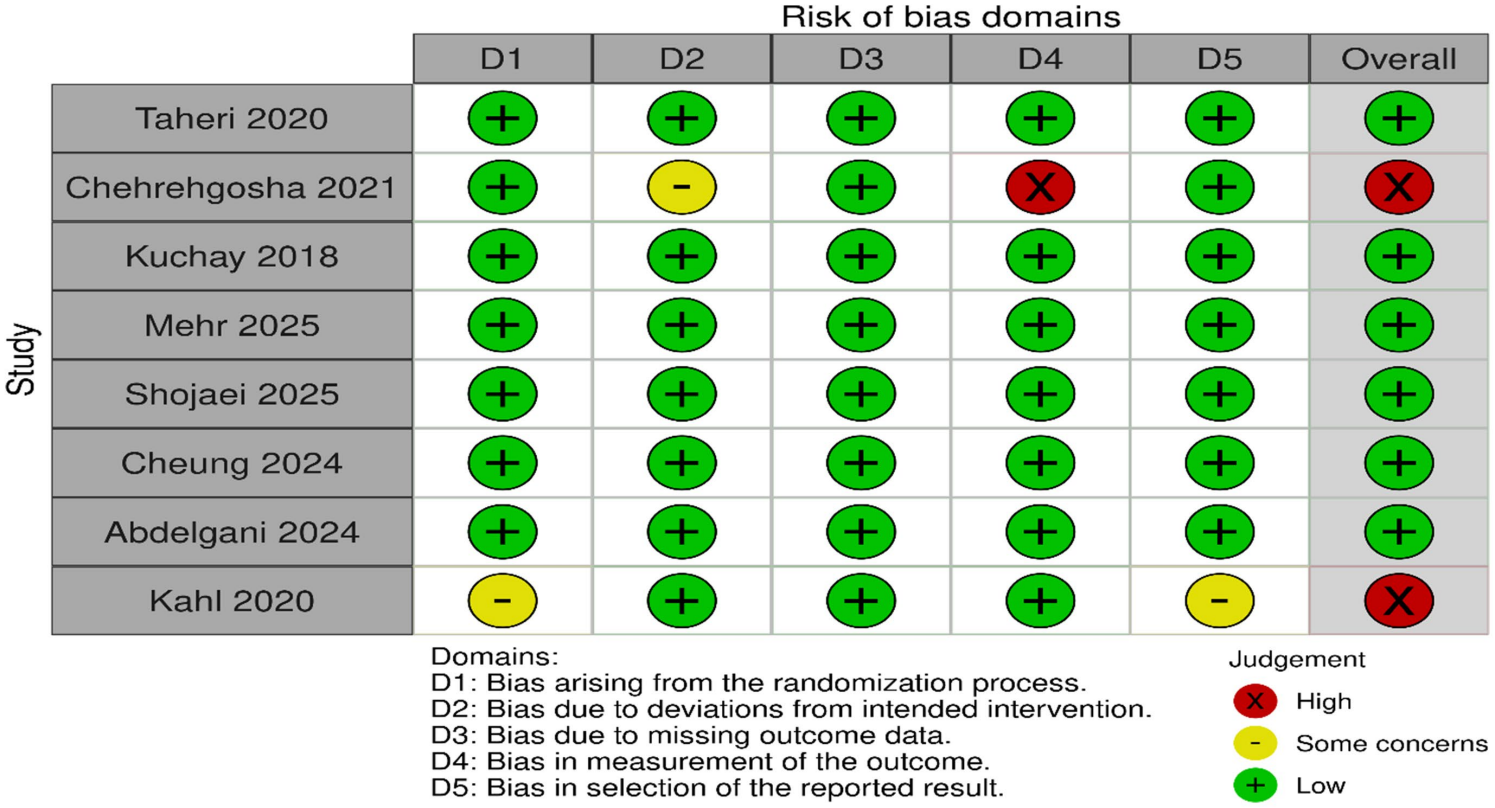
Risk of bias assessment using RoB 2.

According to RoB 2, six of the included RCTs were deemed to have low risk of bias across all domains, while two had high risk of bias (Figure 2).

### Statistical analysis

**Figure 3:**
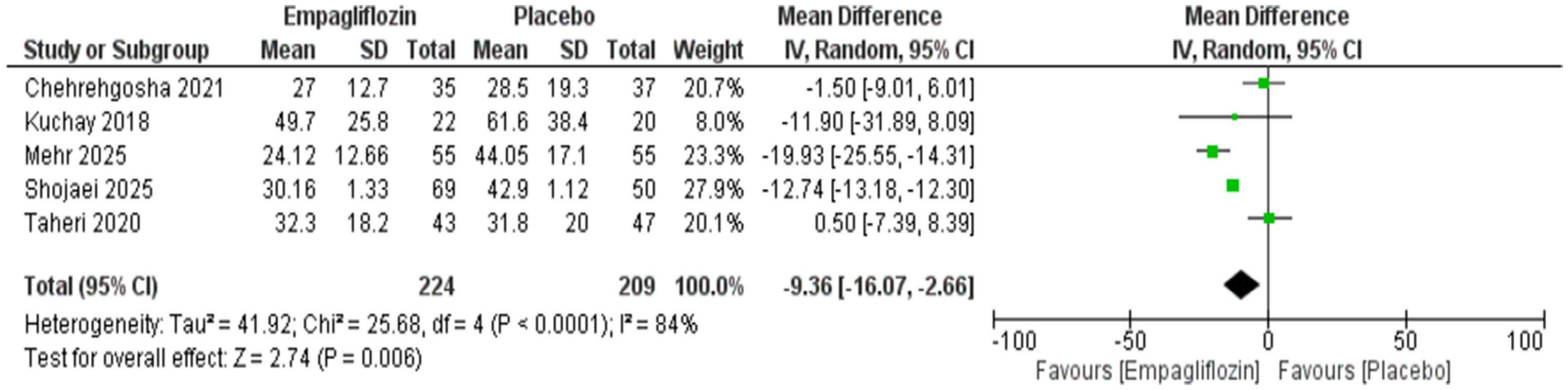
Comparison between empagliflozin and placebo on the effect on ALT.

**Figure 4:**
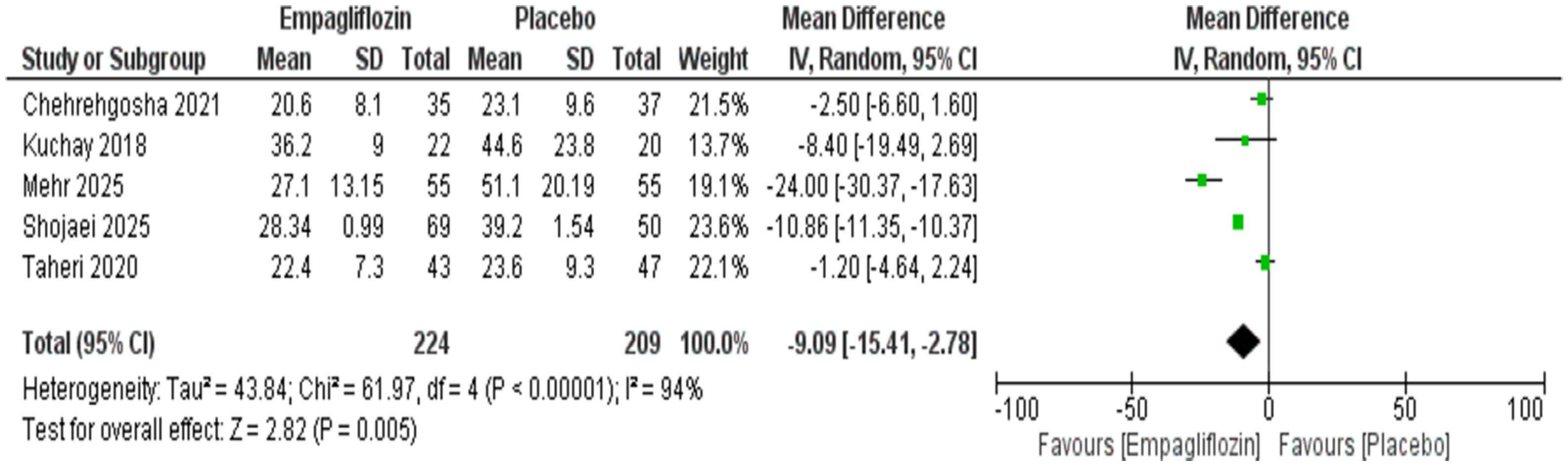
Comparison between empagliflozin and placebo on the effect on AST.

The use of empagliflozin was significantly associated with reduced ALT (MD = −9.36 [95%CI: −16.07, −2.66, *p* = 0.006, I^2^ = 84%, *p* < 0.0001]) and AST (MD = −9.09 [95%CI: −15.41, −2.78, *p* = 0.005, I^2^ = 94%, *p* < 0.00001]) compared with the placebo group (Figures 3, 4).

**Figure 5:**
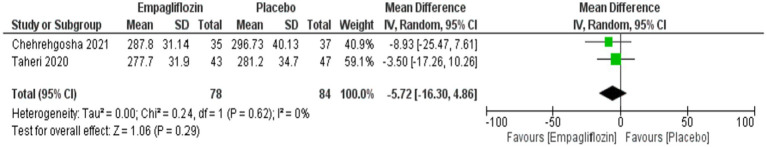
Comparison between empagliflozin and placebo on the effect on CAP.

**Figure 6:**
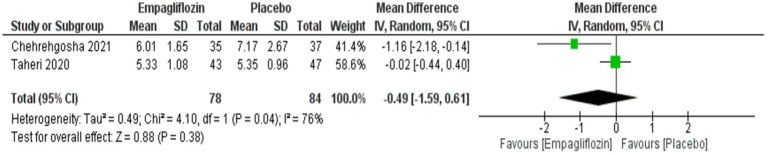
Comparison between empagliflozin and placebo on the effect on LSM.

**Figure 7:**
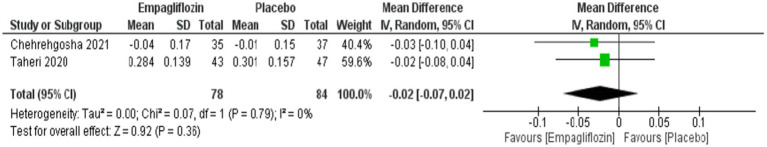
Comparison between empagliflozin and placebo on the effect on APRI.

**Figure 8:**
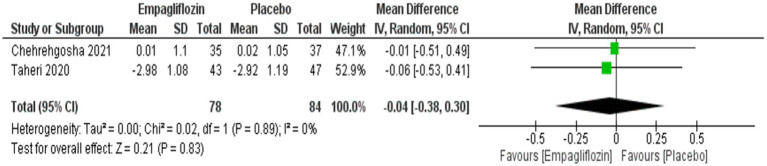
Comparison between empagliflozin and placebo on the effect on NFS.

**Figure 9:**
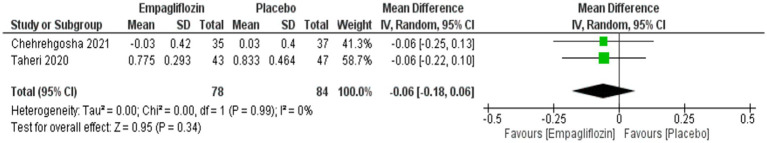
Comparison between empagliflozin and placebo in the effect on FIB-4.

Although empagliflozin was associate with reduced measurements, no significant differences were reached in the comparison between empagliflozin and placebo in CAP with MD = −5.72 (95%CI: −16.3, 4.86, *p* = 0.29), LSM with MD = −0.49 (95%CI: −1.59, 0.61, *p* = 0.38), APRI with MD = −0.02 (95%CI: −0.07, 0.02, *p* = 0.36), NFS with MD = −0.04 (95%CI: −0.38, 0.3, *p* = 0.83) and FIB-4 with MD = −0.06 (95CI: −0.18, 0.06, *p* = 0.34) (Figures 5–9 respectively).

**Figure 10:**
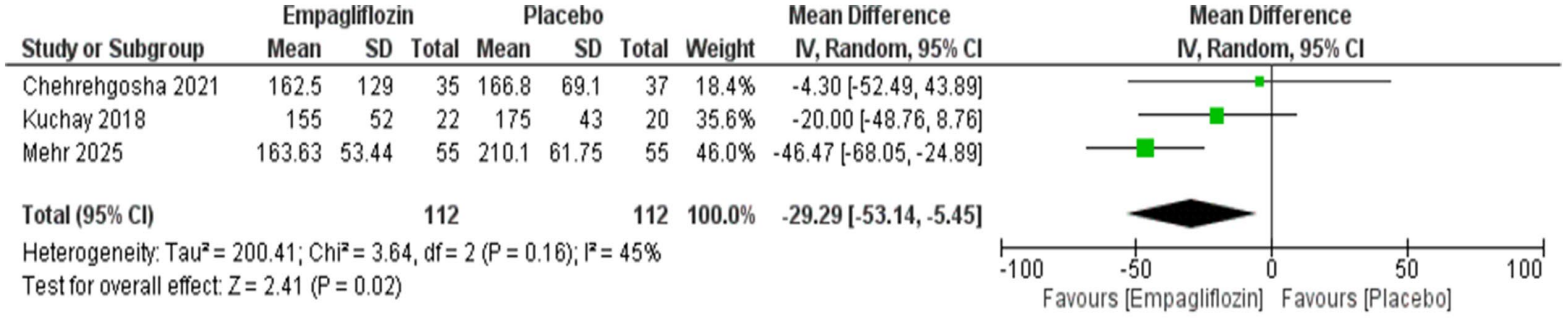
Comparison between empagliflozin and placebo on the effect on triglycerides.

**Figure 11:**
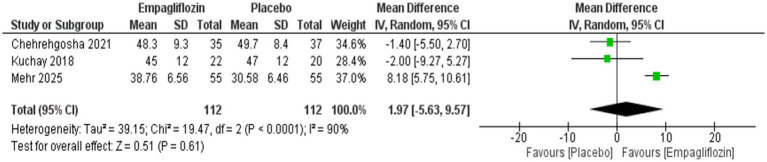
Comparison between empagliflozin and placebo on the effect on HDL.

**Figure 12:**
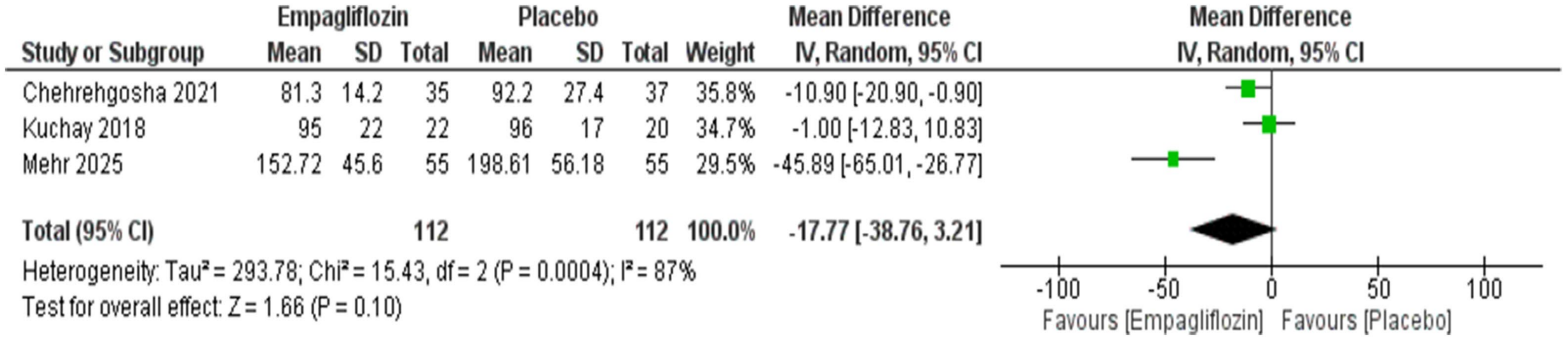
Comparison between empagliflozin and placebo on the effect on LDL.

Empagliflozin was significantly associated with reduced triglycerides compared with placebo, with MD = −29.29 (95%CI: −53.14, −5.45, *p* = 0.02), and I^2^ = 45%, *p* = 0.16 (Figure 10) However, no significant difference was observed between empagliflozin and placebo regarding HDL with MD = 1.97 (95%CI: −5.63, 9.57, *p* = 0.61) and LDL with MD = −17.77 (95%CI: −38.76, 3.21, *p* = 0.10) (Figures 11, 12).

**Figure 13:**
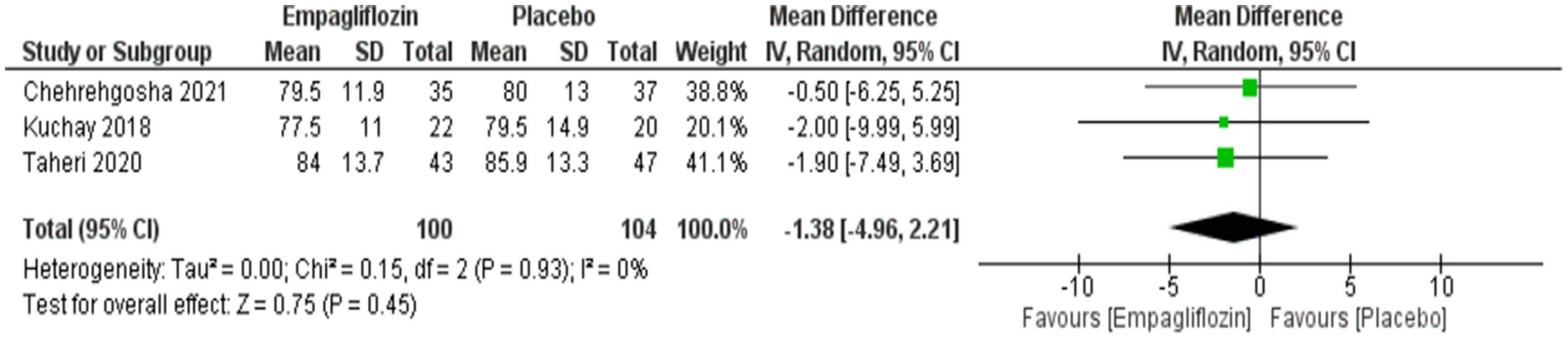
Comparison between empagliflozin and placebo on the effect on weight.

**Figure 14:**
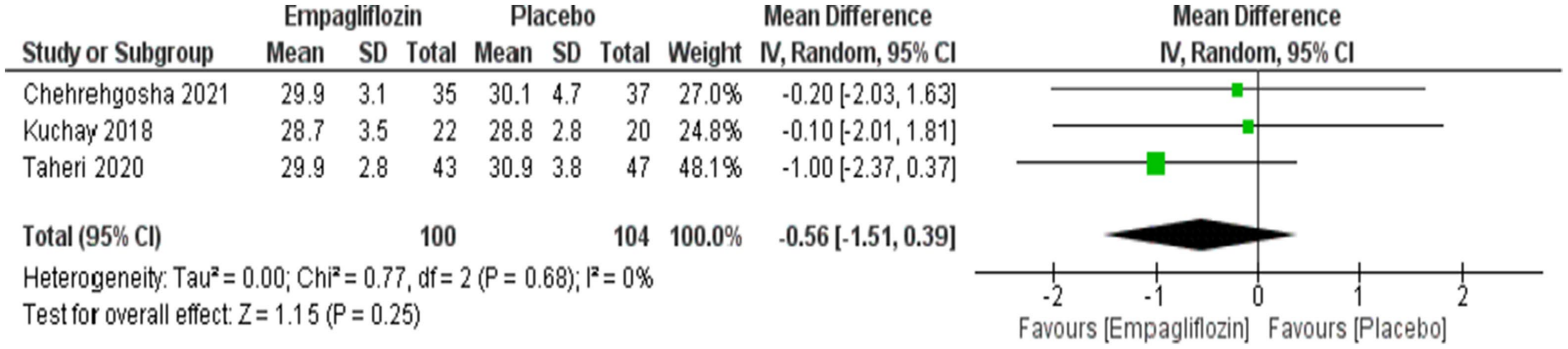
Comparison between empagliflozin and placebo on the effect on BMI.

Although empagliflozin showed lower weight and BMI compared with placebo, no significant difference was obtained between them with MD = −1.38 (95%CI: −4.96, 2.21, *p* = 0.45) for weight and MD = −0.56 (95%CI: −1.51, 0.39, *p* = 0.25) for BMI (Figures 13, 14).

**Figure 15:**
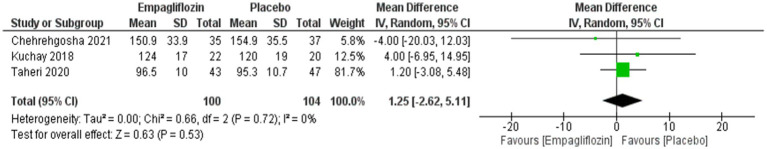
Comparison between empagliflozin and placebo in the effect on FBS.

**Figure 16:**
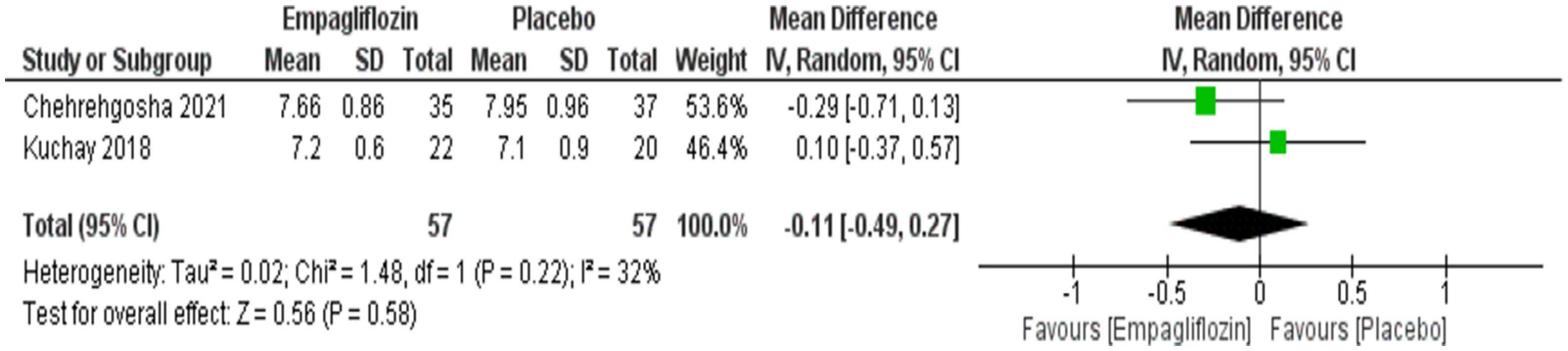
Comparison between empagliflozin and placebo in the effect on HbA1c.

No significant difference was observed between empagliflozin and placebo regarding FBS and HbA1c with MD = 1.25 (95%CI: −2.62, 5.11, *p* = 0.53) and MD = −0.11 (95%CI: −0.49, 0.27, *p* = 0.58), respectively (Figures 15, 16).

### Qualitative synthesis

Three randomized, double-blind, placebo-controlled trials have examined the effects of empagliflozin on hepatic steatosis in different populations, including patients with well-controlled T2D, individuals with MASLD without diabetes, and mixed cohorts comprising both diabetic and non-diabetic participants. All trials quantified hepatic fat content using magnetic resonance spectroscopy or MRI-proton density fat fraction (MRI-PDFF), with treatment durations ranging from 12 to 52 weeks.

In adults with recent-onset, well-controlled T2D, Kahl et al. (24) reported that 24 weeks of empagliflozin 25 mg daily produced a placebo-corrected absolute reduction in liver fat content of −1.8% (95% CI − 3.4 to −0.2; *p* = 0.02), corresponding to a relative reduction of approximately −22% (−36 to −7; *p* = 0.009). These reductions occurred despite no significant improvement in clamp-measured insulin sensitivity, although empagliflozin was associated with a mean placebo-corrected weight loss of −2.5 kg (*p* < 0.001), a fall in uric acid of −74 μmol/L (*p* < 0.001), and a 36% increase in high-molecular-weight adiponectin (*p* < 0.001).

Similar benefits were observed in a 12-week trial that enrolled both diabetic and non-diabetic adults (22). In this study, the absolute change in liver fat content was −2.39% ± 0.79% with empagliflozin compared with +0.91% ± 0.64% with placebo (ANOVA *p* < 0.007). Subgroup analyses revealed consistent effects in participants with T2D (−2.75% ± 0.81% vs. placebo, *p* < 0.05) and without T2D (−1.93% ± 0.78% vs. placebo, *p* < 0.05). Importantly, individuals with higher baseline liver fat (>7%) experienced larger absolute reductions, reaching −5.4% in T2D and −4.17% in non-diabetic participants. Correlation analyses showed that reductions in liver fat were closely linked to both weight loss (*r* = 0.53; *p* < 0.001) and improvements in insulin sensitivity as measured by the Matsuda index (*r* = −0.51; *p* < 0.001). Multivariable models suggested that each standard deviation improvement in insulin sensitivity (≈0.5 units) was associated with a ~ 1.5% absolute reduction in liver fat, and each 3.3 kg weight loss corresponded to a ~ 1.78% reduction, independent of glycemic status. Three participants developed vaginal yeast infections, but all completed the study without discontinuation.

The longest trial, conducted by Cheung et al. (23), enrolled 98 non-diabetic adults with MASLD and treated them with empagliflozin 10 mg or placebo for 52 weeks. At 1 year, empagliflozin achieved a greater median reduction in MRI-PDFF compared with placebo (−2.49% vs. −1.43%; *p* = 0.025). Although steatosis resolution, defined as MRI-PDFF <5%, occurred more frequently in the empagliflozin arm (44.9% vs. 28.6%), this difference did not reach statistical significance (*p* = 0.094). Similarly, the proportion achieving ≥30% MRI-PDFF decline was 49.0% vs. 40.8% (*p* = 0.417). Secondary outcomes demonstrated modest but significant reductions in body weight (−2.7 vs. −0.2 kg) and waist circumference (−2.0 vs. 0 cm), as well as a fall in fasting glucose (−0.3 vs. 0 mmol/L; *p* < 0.05). Liver enzyme responses were less consistent, with no significant difference in the proportion achieving ALT decline ≥17 U/L (16.3% vs. 12.2%; *p* = 0.564). However, serum ferritin declined significantly more with empagliflozin (−126 vs. −22 pmol/L; *p* < 0.05).

## Discussion

### Summary of findings

This systematic review and meta-analysis evaluated the efficacy of empagliflozin in patients with MASLD, including those with and without T2DM. The findings consistently demonstrate that empagliflozin exerts a favorable effect on liver enzyme levels, specifically ALT and AST, suggesting a potential role in improving hepatic inflammation or injury in this patient population. Despite these improvements in liver enzymes, the analysis did not reveal significant differences between empagliflozin and placebo in terms of non-invasive liver fibrosis and steatosis markers, including CAP, LSM, APRI, NFS, and FIB-4, but it is important to consider that the MD was favoring empagliflozin and the sample size was small, which may have failed to reach statistical significance. Regarding metabolic outcomes, empagliflozin was associated with improvements in lipid profile parameters, particularly triglycerides, but showed no significant effect on HDL or LDL levels. Although reductions in body weight and BMI were observed in patients receiving empagliflozin, these changes did not reach statistical significance when compared to placebo, reflecting a modest trend rather than a definitive metabolic benefit. In terms of glycemic control, no notable differences were detected in fasting blood sugar or HbA1c, suggesting that the hepatic benefits observed with empagliflozin may not be entirely mediated through its glucose-lowering effects. Overall, the findings support the hepatic safety and potential liver-related benefits of empagliflozin in patients with MASLD, particularly in terms of enzyme normalization. However, the lack of significant improvements in fibrosis markers and broader metabolic indices highlights the need for further long-term and mechanistically focused studies to better understand its role in liver disease management.

The narrative synthesis showed that empagliflozin consistently lowers hepatic fat compared with placebo across diverse patient populations. The placebo-corrected absolute reductions in liver fat ranged from −1.8% over 24 weeks in T2D, to −2.39% over 12 weeks in mixed populations, and −2.49% over 52 weeks in non-diabetic MASLD. Larger absolute reductions of 4–5% were observed in individuals with higher baseline steatosis. While these effects are modest in absolute terms, they are reproducible across settings and appear clinically relevant when considered alongside associated improvements in body weight, central adiposity, and markers of insulin sensitivity. The benefits seem to be mediated at least partly by weight loss and improved insulin action, although pleiotropic effects such as increased adiponectin and reduced uric acid may also play a role.

### Investigation with previous literature

The current findings are broadly consistent with, yet also build upon, two previously published meta-analyses evaluating the efficacy of empagliflozin in MASLD. Tang et al. (25), who included only three RCTs with a total of 212 participants, reported no significant improvements in hepatic steatosis (CAP), fibrosis (LSM), or liver enzyme levels (ALT and AST), concluding that empagliflozin may not be effective for MASLD management. In contrast, Zhang et al. (14), analyzing four RCTs with 244 participants, demonstrated a significant reduction in AST and LSM scores, along with a modest, non-significant reduction in ALT and CAP. Our meta-analysis extends these findings by incorporating eight RCTs and a larger pooled sample, including the most recent trials conducted in 2025. Notably, our study revealed statistically significant reductions in both ALT and AST levels, reinforcing empagliflozin’s potential hepatoprotective effects. Additionally, a significant decrease in triglyceride levels was observed, which was only marginal in prior analyses. However, similar to Zhang et al. (14) and Tang et al. (25), our findings did not demonstrate statistically significant improvements in non-invasive markers of fibrosis or glycemic control parameters. These discrepancies may be attributed to differences in sample sizes, population characteristics (e.g., diabetic vs. non-diabetic), and the inclusion of more contemporary studies in our analysis. Collectively, the evidence underscores a consistent trend toward hepatic biochemical improvement with empagliflozin, while its impact on structural liver outcomes and metabolic parameters remains inconclusive and warrants further long-term investigation.

Several previous investigations have explored the impact of different SGLT2 inhibitors on patients diagnosed with both T2DM and MASLD (16, 26–30). These studies have consistently demonstrated beneficial effects, with improvements noted in at least one liver enzyme parameter such as ALT or AST (16, 27, 28, 30, 31), as well as reductions in hepatic fat accumulation (16, 27, 30). Notably, the E-LIFT trial evaluated empagliflozin in patients with MASLD and reported a 4% absolute reduction in liver fat content, which was associated with a meaningful improvement in hepatic steatosis (16). However, the study employed magnetic resonance imaging-proton density fat fraction (MRI-PDFF), a sensitive but costly and time-intensive imaging modality that limits routine clinical application (32). In a Japanese cohort, ipragliflozin was found to reduce liver fat in T2DM patients with MASLD, but liver fat was assessed using the fatty liver index, a surrogate marker with limited diagnostic precision (33). Similarly, reductions in hepatic fat content were observed in patients treated with luseogliflozin, using the liver-to-spleen (L/S) attenuation ratio, although this method is considered less accurate for quantifying liver fat (30). Shimizu et al. (27) also reported significant improvements in the CAP among patients receiving dapagliflozin compared to placebo. Additionally, when ipragliflozin was directly compared to pioglitazone, a decrease in the L/S ratio was observed on computed tomography (CT), though the difference between treatment groups was not statistically significant (26).

### Clinical implications

The findings of this meta-analysis carry important clinical implications for the management of MASLD, particularly in patients with coexisting T2DM. The significant reductions in ALT and AST observed in our analysis suggest that empagliflozin may exert hepatoprotective effects, likely through mechanisms related to reduced hepatic inflammation, improved insulin sensitivity, and favorable alterations in lipid metabolism. Empagliflozin inhibits SGLT2 receptors in the renal proximal tubules, leading to glucosuria and reduced plasma glucose. Beyond this renal mechanism, empagliflozin improves hepatic outcomes through weight reduction, improvements in adipose tissue inflammation, modulation of adipokines, reduction of oxidative stress, and decreased hepatic lipotoxicity (34). These effects are especially relevant given the absence of currently approved pharmacological therapies for MASLD, positioning empagliflozin as a promising adjunct in the therapeutic aspect. The observed reduction in triglyceride levels further supports its metabolic benefits, particularly in the context of dyslipidemia frequently seen in MASLD. While improvements in steatosis and fibrosis markers (e.g., CAP, LSM, FIB-4, NFS, and APRI) were not statistically significant, the consistent trend favoring empagliflozin across these outcomes suggests a potential for benefit with longer treatment duration or in specific subgroups of patients. The absence of significant changes in glycemic markers (HbA1c and FBS) in our pooled analysis may reflect the relatively well-controlled diabetic status of participants or the short duration of most trials. Also, this may be attributed to the small sample sizes in these outcomes (HbA1c and FBS) due to limited published data. Clinically, empagliflozin can be considered a viable therapeutic option for patients with MASLD, particularly those with T2DM, where it may serve dual roles in improving glycemic control and mitigating hepatic injury. However, due to limited effects on fibrosis and lack of histological data, it should not yet replace lifestyle interventions or emerging targeted therapies. Instead, it may be most appropriately positioned as part of a comprehensive, multimodal treatment strategy that addresses the metabolic, hepatic, and cardiovascular dimensions of MASLD.

A growing body of evidence suggests that SGLT2 inhibitors, such as empagliflozin, may exert therapeutic effects on MASLD. Preclinical data from animal models of fatty liver disease indicate that SGLT2 inhibition can lead to decreased hepatic fat accumulation, improvements in liver enzyme profiles, and reductions in inflammatory markers (35). These findings are supported by clinical trial results demonstrating that SGLT2 inhibitors contribute to lowering aminotransferases—specifically ALT and AST—as well as improving non-invasive markers of liver fibrosis in patients with MASLD. Although the precise biological mechanisms underlying empagliflozin’s hepatic benefits remain under investigation, several plausible pathways have been proposed. These include enhanced lipid metabolism, improved peripheral and hepatic insulin sensitivity, attenuation of systemic and hepatic inflammation, and modest but consistent weight reduction—all of which are integral components in the pathogenesis and progression of MASLD (36).

Age-related insulin resistance, sarcopenia, and sex-related differences in visceral adiposity may influence the magnitude of hepatic response to empagliflozin. However, the included trials did not report sufficient subgroup data to quantitatively assess these modifiers (37).

The consensus recommends adopting the MASLD/MASH terminology and applying standardized metabolic criteria for diagnosis while prioritizing non-invasive fibrosis assessment, such as FIB-4 and elastography, to guide risk stratification and referral. Management should center on lifestyle modification with targeted weight loss, alongside metabolic therapies including GLP-1 receptor agonists, SGLT2 inhibitors, pioglitazone, and bariatric surgery when appropriate. A multidisciplinary approach is emphasized, with hepatology referral for individuals with suspected advanced fibrosis or inadequate response to conservative and pharmacologic interventions (38).

Guideline implications for MASLD emphasize systematic case-finding and risk stratification in people with cardiometabolic risk (particularly T2D), using simple first-line tests such as FIB-4 followed by elastography or other second-line NITs to decide who needs specialist referral and closer monitoring. Lifestyle modification and targeted weight loss remain the cornerstone of management, but guidelines now explicitly support using evidence-based metabolic therapies to treat comorbidities and improve liver outcomes — notably GLP-1 receptor agonists, SGLT2 inhibitors, and pioglitazone as options for patients with T2DM or obesity, while new MASH-directed drugs (e.g., the THR-*β* agonist resmetirom) are recommended where licensed for selected patients with F2–F3 fibrosis. Clinicians should adopt a multidisciplinary approach (primary care, endocrinology, hepatology, nutrition/behavioral support), monitor treatment response with agreed NIT-based algorithms rather than relying solely on transaminases, and prioritize long-term, individualized care plans that balance metabolic, hepatic, and cardiovascular risks (39, 40).

### Strengths and limitations

This meta-analysis offers several methodological strengths that enhance the reliability of its findings. It represents the most up-to-date and comprehensive synthesis of RCTs assessing the effects of empagliflozin in patients with MASLD, incorporating eight RCTs with a total of 672 participants. The inclusion of both diabetic and non-diabetic populations improves the generalizability of the findings across varying clinical profiles. A major strength of this work lies in the breadth of outcomes analyzed, encompassing not only hepatic enzyme levels and steatosis markers, but also a wide range of non-invasive fibrosis indices and metabolic parameters. The rigorous approach taken to study selection, data extraction, and risk of bias assessment following PRISMA guidelines and using the Cochrane RoB 2.0 tool further reinforces the methodological quality of this review.

Nonetheless, several limitations should be acknowledged, primarily from a research design and evidence quality standpoint. First, all included studies were of relatively short duration, which may be insufficient to observe meaningful changes in hepatic fibrosis or long-term liver-related outcomes. Second, considerable heterogeneity was observed across certain outcomes, and while a random-effects model was applied, the variability in study design, baseline characteristics, and outcome reporting introduces potential bias. Heterogeneity may also stem from differences in MASLD severity, diabetic status, imaging modalities, empagliflozin dose, and treatment duration. However, it was present in small number of outcomes, and due to the limited sample size and number of published studies, subgroup or sensitivity analysis could not resolve it. Third, the geographic concentration of studies in Iran may affect the external validity of the results, especially in underrepresented populations such as those in Western countries. Fourth, some studies did not report quantitative outcomes used in the current meta-analysis, so we conducted a narrative synthesis approach. Fifth, the included outcomes did not report adverse events or histological parameters. Not registering the protocol of the meta-analysis is also a limitation that should be acknowledged. Finally, the relatively small sample sizes in individual trials raise the possibility of type II errors and may reduce the precision of pooled effect estimates. Also, the small sample size in most of the outcomes due to the limited number of published studies may limit the statistical power.

### Recommendations

To address these gaps, future research should prioritize long-term, adequately powered RCTs with follow-up durations extending beyond 6 months and incorporate liver histology as a primary endpoint. The inclusion of multicenter trials conducted across diverse geographic and ethnic populations will help enhance generalizability. Furthermore, standardization in outcome measures and reporting practices across trials would facilitate more consistent data synthesis in future meta-analyses. Investigations should also focus on evaluating the mechanistic pathways through which empagliflozin may exert hepatic effects, using biomarker-based and imaging-based approaches. Finally, comparative effectiveness research assessing empagliflozin against other emerging therapies for MASLD whether used alone or in combination could provide valuable insights into optimizing treatment strategies in future clinical trials.

## Conclusion

Empagliflozin significantly improves hepatic enzyme levels (ALT and AST) and reduces triglyceride concentrations in patients with MASLD, suggesting a favorable effect on liver inflammation and lipid metabolism. However, the agent did not show statistically significant effects on non-invasive markers of steatosis or fibrosis, body weight, or glycemic indices within the short treatment durations studied. While these findings support the hepatic safety and partial metabolic benefit of empagliflozin, the lack of histological data and long-term outcomes underscores the need for future high-quality RCTs. These studies should incorporate liver biopsy endpoints, longer follow-up periods, and diverse patient populations to fully elucidate the therapeutic potential of empagliflozin in the treatment and progression of MASLD.

## Data Availability

The original contributions presented in the study are included in the article/supplementary material, further inquiries can be directed to the corresponding author.
